# A new inactivated *Tritrichomonas foetus* vaccine that improves genital clearance of the infection and calving intervals in cattle

**DOI:** 10.3389/fvets.2022.1005556

**Published:** 2022-10-06

**Authors:** Luis Miguel Ortega-Mora, Roberto Sánchez-Sánchez, Silvia Rojo-Montejo, Alicia Román-Trufero, Dolores Montenegro-Gregorio, Eugenia Puentes-Colorado, Alberto Parra-Romero, Javier Regidor-Cerrillo, Koldo Osoro, Esther Collantes-Fernández

**Affiliations:** ^1^SALUVET, Animal Health Department, Faculty of Veterinary Sciences, Complutense University of Madrid, Madrid, Spain; ^2^SALUVET-Innova S.L., Faculty of Veterinary Sciences, Complutense University of Madrid, Madrid, Spain; ^3^Regional Service for Research and Agri-Food Development (SERIDA), Villaviciosa, Spain; ^4^CZ Vaccines S.A.U., ZENDAL Group, Pontevedra, Spain

**Keywords:** Bovine trichomonosis, *Tritrichomonas foetus* vaccine, cattle, immunogenicity, safety, efficacy

## Abstract

Bovine trichomonosis is a sexually transmitted disease that is a primary cause of early reproductive failure in cattle. The aim of the present study was to develop a vaccine formulation based on *Tritrichomonas foetu*s trophozoites inactivated by lyophilization and Quil-A-adjuvanted. The safety, immunogenicity and efficacy of this new vaccine formulation (Trichobovis^®^) administered by two routes (subcutaneous: SC, and intravulvar: IVU) were compared with a commercial vaccine (TrichGuard^®^) in a well-established experimental bovine model of genital *T. foetus* infection. The new vaccine was considered safe in cattle because only mild local reactions were found in the vaccination area, which disappeared 3 weeks after administration. Cows immunized with Trichobovis cleared the infection faster than the non-immunized/challenged group (27–28 vs. 60 days; *P* < 0.05). Not significant differences were observed with the commercial vaccine respect to the positive control group, or between SC and IVU routes. The new vaccine stimulated high serum anti-*T. foetus* IgG and genital IgA levels and generated an IgG booster effect similar to TrichGuard. IgA levels were associated with significantly earlier genital clearance of *T. foetus* in cows immunized with Trichobovis by SC route (G1A) or TrichGuard (G2). The strongest association was found in the group G1A on day 14 post-infection (p.i.) (*r* = −0.74) and in G2 on day 35 p.i. (*r* = −0.71). The efficacy of vaccination using Trichobovis on the reproductive performance was also investigated under field conditions in a herd where *T. foetus* was present. The calving intervals were significantly reduced by 45.2 days (*P* < 0.05), calves were born 28 days earlier (*P* < 0.05) and an increase of 8.7% in the calving rate (*P* > 0.05) was observed in the vaccinated group. These results demonstrate that Trichobovis improved the reproductive performance under field conditions in herds where *T. foetus* infection is present.

## Introduction

Bovine trichomonosis caused by *Tritrichomonas foetus* is a sexually transmitted disease that is included in the list of diseases of the World Organization for Animal Health (OIE). Bovine trichomonosis is a primary cause of early reproductive failure in cattle, and it leads to significant financial losses (1–3). Bovine trichomonosis is endemic worldwide in herds managed under extensive conditions and using natural services for mating (4). The disease is associated with lowered fertility in infected herds. Usual signs in the herd include return to oestrus after mating, many services per conception, poor pregnancy rates, long calving intervals and calf crop reduction. Therefore, the calving season is also spread out, and this causes batches of calves of different ages with a wide variation in weaning weights, which has a significant negative impact on the productivity in beef cattle herds (5–7).

The parasite is localized in the preputial cavity of bulls and the genital tract of females, and it is transmitted during coitus. *T. foetus* infection is asymptomatic in males, and it does not affect semen quality or sexual behavior; however, the bull is a lifelong asymptomatic carrier and transmitter of the disease (8, 9). Parasite multiplication causes inflammation of the reproductive tract, including cervicitis and endometritis, in cows or heifers following infection at mating. Therefore, *T. foetus* infection causes embryonic death or early abortion from 42 days of gestation, with a peak loss at 50–70 days of gestation. Infection in females is generally self-limiting and disappears after 2 and 4 months, i.e., after three cycles. The immunity that develops is not permanent and generally lasts for approximately 6 months; then the female is susceptible to re-infection (9–11).

There are no effective drugs to control bovine trichomonosis, and control relies primarily on testing and culling policy (12, 13), which is an effective measure to restore reproductive efficiency and productivity (7). There is evidence of a re-emergence of bovine trichomonosis in extensive husbandry conditions in Australia (14, 15), South Africa (16), South America (17–19), EEUU (13, 20), and Europe (3, 4). Programs for prevention and control are partially successful because risk factors (communal pastures, the lack of diagnostic tests before the mating season and a high proportion of bulls older than 3 years) cannot be avoided (7). Vaccination may be a feasible control measure to reduce losses due to bovine trichomonosis (21). Vaccines are only indicated for cows because they do not induce protection in bulls (22). Vaccines will not prevent infection in most cases, but they reduce the clearance time of *T. foetus* from the reproductive tract before fetal loss occurs and improve pregnancy rates by decreasing the duration of endometritis (22–26). However, commercial vaccines are only available in some regions of the world (9).

Therefore, a new vaccine for controlling the effects of *T. foetus* in cattle that improves on the efficacy of commercial vaccines is desirable. The present study developed an inactivated vaccine (Trichobovis^®^) that is safe and capable of reducing *T. foetus* infection in cattle. Whole parasite trophozoites were used as antigen, including an inactivation procedure of the microorganism (lyophilisation) that better guarantee antigen preservation and parasite inactivation. The safety, immunogenicity and efficacy of this new vaccine formulation were compared with a commercial vaccine (TrichGuard^®^) in a well-established experimental bovine model of genital *T. foetus* infection. Different routes of administration were also evaluated in the same model. The efficacy of Trichobovis and the selected route of administration was evaluated by investigating the reproductive performance in a beef cow-calf herd under field conditions to guide farmers' decisions on vaccination against bovine trichomonosis.

## Materials and methods

### *Tritrichomonas foetus* isolates and culture

Trophozoites for vaccine production and challenge were cultured *in vitro* in modified Diamond's medium at 37 °C (27). The modified medium was prepared according to the following formula: Milli-Q water (79.2%), CASO-Broth culture media (2%) (Reference 22098, Sigma-Aldrich, Madrid, Spain), yeast extract (1%) (Reference 288610, Thermo Fisher Scientific, Waltham, MA, USA), maltose (0.5%) (Reference 1059100500, Sigma-Aldrich, Madrid, Spain), L-cysteine hydrochloride (0.1%) (Reference C7352, Sigma-Aldrich, Madrid, Spain), L-ascorbic acid (0.02%) (Reference A92902, Sigma Aldrich, Madrid, Spain), dibasic potassium phosphate (anhydrous) (0.08%) (Reference S5136, Sigma-Aldrich, Madrid, Spain), monobasic potassium phosphate (anhydrous) (0.08%) (Reference S5011, Sigma-Aldrich, Madrid, Spain), sterile lamb serum (5–10%) (Reference 16070096, Thermo Fisher Scientific, Waltham, MA, USA), penicillin-streptomycin (1%) (Reference 15140122, Thermo Fisher Scientific, Waltham, MA, USA), streptomycin sulfate (500 mg/mL) (1%) (Reference A1852.0025, VWR, Radnor, PA, USA) and amphotericin B (250 μg/mL) (4%) (Reference A2942, Sigma-Aldrich, Madrid, Spain).

Isolate no. 96 of *T. foetus*, which was obtained from the smegma of a Spanish infected bull (28), was used for vaccine production. In order to avoid the existence of different *T. foetus* cell populations in the original isolate, a pure strain containing a single cell was obtained. The single cell isolation was performed *via* limit dilution in tubes to create serial decimal dilutions in modified Diamond medium from 10^5^ trophozoites/tube to 1 trophozoite/tube (29). Trophozoites were grown in modified Diamond's medium at 37 °C and collected in the logarithmic phase of growth (48–72 h). The cultures were centrifuged at 2,000 × g for 10 min, the supernatant was removed, and the trophozoite pellets were washed 5 times in phosphate-buffered saline (PBS). The number of total viable purified trophozoites was determined by counting in a Neubauer chamber. All batches of purified trophozoites showed a viability > 95%. The washed cells were resuspended in 2 mL of PBS with 0.1% protease inhibitor (Reference P2714, Sigma-Aldrich; 1 μL/mL) to a final concentration of 1× 10^8^ whole cells/mL, dispensed into sterile vials and stored at −80 °C until lyophilization.

Parasites (isolate no. 14) for challenge in study no. 1 (see Section Study no. 1) were obtained from the smegma of a Spanish infected bull belonging to a different herd from where vaccine isolate (no. 96) was obtained and where a significant deleterious effect on reproductive efficiency was found as a consequence of *T. foetus* infection (7). Trophozoites were grown in modified Diamond's medium at 37 °C and collected in the logarithmic phase of growth (48–72 h), as described above. The number of total viable purified trophozoites was determined by counting in a Neubauer chamber (>99% viability).

### Vaccine formulation

For parasite inactivation, frozen trophozoites (prepared as described above) were lyophilised at 0.050 mbar/−80 °C for 24 h using a Telstar CRYODOS lyophiliser (Telstar, Barcelona, Spain). The vaccine formulation was dispensed in sterile bottles, and no microbial contamination was recorded (data not shown). To assess whether the lyophilisation was capable of inactivating trophozoites, 5×10^7^ lyophilised trophozoites were resuspended in 2 mL of PBS and cultured in 10 mL of modified Diamond medium for 7 days at 37 °C (this experiment was performed in quadruplicate). No viable trophozoites were observed in *in vitro* culture during the week following inoculation. The vaccine antigen (lyophilised trophozoites of *T. foetus* with protease inhibitor) was mixed with 750 μg of Quil-A adjuvant filtered through a 0.22 μm membrane following the manufacturer's recommendations (Accurate Chemical and Scientific Corporation, Westbury, NY, USA). Specifically, 0.2 μL of Quil-A was resuspended in 10 mL of sterile PBS and used as a diluent for lyophilised trophozoites. The final volume of 2 mL per dose contained 5×10^7^ lyophilised trophozoites, 0.4 μL of the protease inhibitor and 750 μg of Quil-A adjuvant.

Cattle from study no. 2 (see Section Study no. 2.) were vaccinated with the lyophilised freeze-dried trophozoite antigen-based formulation of *T. foetus* (Trichobovis^®^) manufactured by CZ Veterinaria S.A. according to the production method described in the dossier registered by the Spanish Agency for Medicines and Health Products (AEMPS) (AEMPS authorization: 391/ECV and PIV: 181/PIV).

### Safety, efficacy and immunogenicity of Trichobovis in cattle

Two different studies were performed to test the new vaccine in cattle. Study no. 1 used a well-established experimental bovine model of genital *T. foetus* infection (26, 30, 31) to evaluate the safety, immunogenicity and efficacy of Trichobovis under controlled conditions. Safety was assessed by studying the local and systemic reactions, including rectal temperature recording, immune response by analyzing IgG in serum and IgA in cervical-vaginal mucus (CVM) samples; and efficacy by analyzing the time for parasite clearance in the female genital tract. Trichobovis was tested in parallel with a commercial vaccine (TrichGuard; Boehringer Ingelheim Vetmedica, Inc., St. Joseph, MO, USA). Study no. 2 investigated systemic adverse effects attributable to vaccination and the efficacy of the new vaccine on reproductive performance under field conditions in a herd where *T. foetus* infection was present.

#### Study no. 1

##### Animals

The Animal Research Ethics Committee (reference number PROAE 13/2014) of the Community of Principado de Asturias, Spain approved all protocols involving animals, following the proceedings described in Spanish and EU legislations (Law 32/2007, R.D. 53/2013, and Council Directive 2010/63/EU). All animals were handled in strict accordance with good clinical practices, and all efforts were made to minimize suffering.

The experimental animals consisted of 63 nulliparous Holstein breed heifers that were 24 to 36 months old and housed in the cattle facilities belonging to the Regional Service for Agri-food Research and Development (SERIDA) (Villaviciosa, Asturias, Spain). Animals were seronegative for *Neospora caninum*, infectious bovine rhinotracheitis (IBR) virus, bovine viral diarrhea (BVD) virus and *Leptospira* (*L. hardjo* and *L. pomona*), negative for the presence of the BVD virus, negative for brucellosis and tuberculosis, and vaccinated against the common viral respiratory/reproductive pathogens: BVD, IBR, parainfluenza Type 3 (PI-3), and bovine respiratory syncytial virus (VRSB) using HIPRABOVIS^®^4 vaccines (Laboratorios Hipra, Madrid, Spain).

##### Experimental design, vaccination, challenge and sample collection

Animals were randomly allocated into six groups (Table 1). Groups 1A and 1B (G1A and G1B) consisted of animals immunized with Trichobovis and Group 2 (G2), which received the whole inactivated *T. foetus* vaccine TrichGuard according to the manufacturer's recommendations. The other groups consisted of non-vaccinated/challenged animals (G3) and non-vaccinated/non-challenged animals (G4A and G4B). The animals were first immunized (day−42 of the study) *via* the subcutaneous (SC) route in the neck (right side) with Trichobovis (G1A and G1B) or the commercial vaccine (G2). Animals were subjected to a booster on day−21 via the SC route (G1A and G2) in the neck (left side) or intravulvar (IVU; G1B) route. The dose used in each administration was 2 mL (5×10^7^ lyophilised trophozoites) of the vaccine for each animal. Synchronization of the oestrus was achieved by two intramuscular doses (500 μg) of prostaglandin F2 analog (Estrumate, Schering-Plow, Germany) with an interval of 11 days. On day 0 of the experiment (day of oestrus), cows from G1A, G1B, G2, and G3 were challenged intravaginally via inoculation of motile 2×10^6^ trophozoites (>99% viability) of a bovine isolate of *T. foetus* (isolate no. 14) into the fornix using a Cassou pipette.

**Table 1:** Experimental design and local reaction rates of the study no. 1 after the first and second immunisations.

**Group**	**Vaccine**	**Immunization route**	**Challenge**	**Local reaction rates**	
				**Immunization I**	**Immunization II**
**G1A** (n = 11)	Trichobovis^®^	SC/SC	2 × 10^6^	72.7%	100%
**G1B** (n = 11)	Trichobovis^®^	SC/IVU	2 × 10^6^	72.7%	100%
**G2** (n = 17)	TrichGuard^®^	SC/SC	2 × 10^6^	88%	100%
**G3** (n = 18)	PBS	SC/SC	2 × 10^6^	ND	ND
**G4A** (n = 3)	PBS	SC/SC	0	0%	0%
**G4B** (n = 3)	PBS	SC/IVU	0	0%	0%

SC, subcutaneous; IVU, intravulvar; ND, not determined.

Immunized/challenged groups: Trichobovis via subcutaneous (SC; G1A) or intravulvar (IVU; G1B) routes, TrichGuard by SC route (G2). Non-immunized/challenged animals: G3. Non-immunized/non-challenged groups received PBS injections via SC (G4A) or via IVU (G4B) routes. All challenged cows were inoculated intravaginally with 2 × 10^6^ *T. foetus* trophozoites and non-challenged cows with PBS. Fractions represent animals with any local reaction/animal tested.

Animals were observed during the first 2 h after each immunization for the assessment of the presence of anaphylactic shock then daily to monitor serious adverse reactions (i.e., death) or other clinical signs. Rectal temperatures were also recorded daily within the first week post-immunization and at least twice weekly until the third week post-immunization. Rectal temperatures above 39.5 °C were considered a clinical sign of febrile reaction. Tissue injection-site reactions were evaluated to detect soft or firm swellings, abscesses, or ulcers. The diameter of any swelling was measured using a caliper, and the maximum thickness of any swelling was estimated. If a swelling was irregularly shaped (i.e., not round), the largest dimension was recorded as the diameter. Tissue injection-site reactions were measured daily for 10 days and on days 14, 17, and 20 post-immunization.

The efficacy was evaluated by culturing cervical-vaginal mucus (CVM) samples (see Section Protozoal detection in CVM samples) taken every 7 days (after the intravaginal challenge) until the end of the study (day 112 post-infection, p.i.). CVM samples were collected from the anterior portion of the vagina and cervix using a Cassou pipette.

The humoral immune response induced against *T. foetus* was performed *via* the analysis of IgG levels in sera and IgA in CVM from the different groups. For the IgG study (see Section Analysis of IgG responses in serum), blood samples were collected *via* tail vein puncture in 10 mL vacutainer tubes without anti-coagulant (Terumo Europe, Madrid, Spain). Blood samples were taken every 10 days from the first immunization to the challenge then weekly from the challenge until day 112 p.i. Serum samples were recovered after centrifugation at 1,000 × *g* for 10 min and stored at −80 °C until IgG analysis. For IgA analysis (see Section Analysis of IgA responses in CVM), CVM samples were collected every 10 days from the first immunization to the challenge, every 7 days from intravaginal challenge until week no. 9 p.i. then every 2 weeks until week no. 13 p.i. CVM samples were taken from the anterior portion of the vagina and cervix using a Cassou pipette. Approximately 1 mL of each CVM sample was diluted with 2 mL PBS and stored at −80 °C until IgA analysis.

#### Study no. 2

This field study was performed according to the protocol with reference TRIVAC-FR-RD-1 authorized by the AEMPS with authorization number 391/ECV and PIV reference 181/PIV. All interactions with the animals in this study were part of routine farm management where the researcher's involvement was limited to vaccination, observation and data recording in routine farm processes.

##### Study area and herd

The effect of the new vaccine formulation (Trichobovis) on reproductive performance was studied in a suckler herd in which *T. foetus* was detected in herd bulls (*n* = 2) using culture and PCR (3). All animals in the studied herd were from the “Asturiana de la Montaña” breed, which is representative of the sustainable mountain pastoral systems in Spain and is characterized by the management of cattle in fixed periods, which is marked by the start and end of summer, with grazing in communal mountain pastures. Natural mating of “Asturiana de la Montaña” cattle occurs in the spring in the meadows close to the holding and mountain communal pastures in summer. Cattle begin descending from the mountains to the farm in autumn, and births occur between winter and spring (January–June; spring calving herd). This farm was located in areas around “Picos de Europa” National Park in the eastern part of Asturias (north-western Spain), where bovine trichomonosis is endemic (10–40% herd prevalence data) based on our published data (3, 7, 28).

The sanitary status of the animals included screening for the absence of specific antibodies against *N. caninum* in the cows, absence of BVD virus antigen and no amplification of *Campylobacter fetus venerealis* DNA in smegma samples from the herd bulls by a PCR targeting the insertion element ISCfe1 (32). The analytical performance of this PCR assay was initially evaluated in our laboratory (33). The selected farm was also certified as free of bovine tuberculosis and brucellosis. The herd was vaccinated against common viral reproductive-respiratory pathogens (BVD, IBR, PI-3 and VRSB) using HIPRABOVIS^®^4 (Laboratorios Hipra, Madrid, Spain). The bulls (*n* = 2) had a *T. foetus*-positive result (by culture and PCR) 4 months prior to the mating period.

##### Experimental design and data collection

The study design was a double-blinded randomized study with two groups. A total of 46 cows from the same herd, older than 32 months with the same epidemiological and management conditions, were divided into two groups: Group A (*n* = 23) was treated with Trichobovis (vaccinated group), and Group B (*n* = 23) was treated with a placebo (0.002% sucrose in PBS) and was referred as the non-vaccinated group. The animals were first immunized *via* the SC route in the neck with Trichobovis followed by a booster on day 21 using the SC route. The dose used in each administration was 2 mL of Trichobovis vaccine or placebo for each animal. Placebo-treated cows received the same injections as vaccinated animals. Animals were observed after each vaccination for systemic adverse effects attributable to vaccination, as described in study no. 1. Abortigenic, teratogenic, or negative effects on offspring were recorded. Cattle were exposed to infection with *T. foetus* by natural mating with the naturally infected bulls of the herd (*n* = 2) throughout a 120-day mating period. The efficacy of Trichobovis on reproductive performance was investigated by collecting the calving dates at the end of the calving season (June 30^th^) and determining the following parameters: *(i)* calving interval; *(ii)* calving dates and *(iii)* percentage of calves born. Calving intervals were calculated as the number of days between two consecutive calving dates. Calving percentages were calculated as number of calved cows divided by the number of cows that participated in the study.

### Protozoal detection in CVM samples

Culture of *T. foetus* was attempted from all CVMs collected until day 112 p.i. from heifers of study no. 1. Approximately 1 mL of each collected sample was immediately suspended in 2 mL of modified Diamond culture medium, incubated at 37 °C and observed daily under an optical microscope (200× screening; 400× confirmation) for 7 days. Samples were classified as positive based on the presence of motile *T. foetus* trophozoites.

### Analysis of IgG responses in serum

*T. foetus*-specific IgG antibody levels were measured in sera from cows of study no. 1 using an in-house indirect enzyme-linked immunosorbent assay (ELISA) as follows. Soluble antigen, prepared according to (34), was used to coat 96-well microtiter plates (Nunc Maxisorp flat bottom, ThermoFisher Scientific, Waltham, MA, United States). For this step, 100 μL/well of *T. foetus* soluble antigen containing 0.2 μg of protein (quantified using the Bradford method) diluted in carbonate buffer (100 mM, pH 9.6) was incubated overnight at 4 °C. Non-specific binding was blocked by adding 300 μL of a blocking solution (5% horse sera diluted in PBS containing 0.05% Tween 20). After 2 h of incubation at room temperature, the plates were washed four times with PBS containing 0.05% Tween 20 (PBS-T). Serum samples were diluted 1:100 in blocking solution, and 100 μL of this dilution was added to each well and incubated for 1 h at 37 °C. Samples of the reference positive and negative control sera were included in each plate. After four washes in PBS-T, 100 μL of goat anti-bovine IgG (Thermo Fisher Scientific, Massachusetts, MA, USA) diluted 1:8,000 in PBS-T was added and incubated for 1 h at 37 °C. Plates were washed as described above before the addition of 100 μL per well of ABTS substrate (Sigma-Aldrich, Madrid, Spain). The reaction was stopped after 15 min at room temperature by the addition of 100 μL per well of a solution of 0.3 M oxalic acid, and the optical density (OD) was read at 405 nm (OD405). For each plate, the OD values were converted into a relative index percent (RIPC) using the following formula: RIPC = (OD405 sample - OD405 negative control)/(OD405 positive control - OD405 negative control) × 100.

### Analysis of IgA responses in CVM

Samples of CVM from cows of study no. 1 were diluted 1:2 in PBS and homogenized under ice-cooling using a Polytron^®^ PT-1600E (Kinematica AG, Malters, Switzerland) homogeniser and centrifuged at 15,000 × *g* for 20 min at 4 °C, and the supernatants were stored at −20 °C until use (30). *T. foetus*-specific IgA antibody levels were measured in CVM supernatants similarly to the in-house *T. foetus* IgG ELISA described above. Briefly, soluble antigen, prepared according to (34), was used to coat 96-well microtiter plates (Nunc Maxisorp flat bottom, ThermoFisher Scientific, Waltham, MA, United States). For this step, 100 μL/well of *T. foetus* soluble antigen containing 0.2 μg of protein (quantified using the Bradford method) diluted in carbonate buffer (100 mM, pH 9.6) was incubated overnight at 4 °C. Non-specific binding was blocked by adding 300 μL of a blocking solution (5% horse sera diluted in PBS containing 0.05% Tween 20). After 2 h of incubation at room temperature, the plates were washed four times with PBS containing 0.05% Tween 20 (PBS-T). CVM supernatant samples prepared as described previously were diluted 1:50 in blocking solution, and 50 μL of this dilution was added to each well and incubated for 1 h at 37 °C. Samples of the reference positive and negative control CVM were included in each plate. After four washes in PBS-T, 50 μL of rabbit anti-bovine IgA (Fortis Life Sciences, Massachusetts, MA, USA) diluted 1:10,000 in PBS-T was added and incubated for 1 h at 37 °C. Plates were washed as described above before the addition of 50 μL per well of ABTS substrate (Sigma-Aldrich, Madrid, Spain). The reaction was stopped after 15 min at room temperature by the addition of 50 μL per well of a solution of 0.3 M oxalic acid, and the OD was read at 405 nm (OD405). For each plate, the OD values were converted into a relative index percent (RIPC) using the following formula: RIPC = (OD405 sample - OD405 negative control)/ (OD405 positive control - OD405 negative control) × 100.

### Statistical analysis

A repeated measures (two-way) ANOVA test was performed to compare rectal temperatures, local reaction sizes and RIPC values of IgG and IgA. When statistically significant differences were observed, pairwise comparisons between vaccinated and control groups were analyzed using the Bonferroni post-test. Differences in parasite genital infection length were evaluated using one-way ANOVA followed by Bonferroni post-test to compare vaccinated groups vs. the positive control group. The Kaplan-Meier survival method was used to estimate the percentage of animals that cleared the parasite from the genital tract at each time point (days p.i.), and survival (i.e., clearance) curves were compared using the log-rank statistical test. To evaluate whether IgA levels correlated with the duration of genital infection, regression analysis on the different days p.i. of the study was performed to determine the Pearson correlation coefficient (*r*). Calving intervals and calving date means were compared between Groups A and B using Student's *t* test. The percentages of calves born were compared using the chi-square test or Fisher's exact test. Statistical significance for all analyses was established with *P* < 0.05. All statistical analyses were performed using GraphPad Prism 7 v.7.03 (San Diego, CA, USA) software.

## Results

### Vaccine safety

No systemic reactions, such as the presence of anaphylactic shock or serious adverse events (i.e., death attributable to vaccination), were observed in animals from study no. 1 or study no. 2. No abortigenic, teratogenic or negative effects on offspring were found in animals from study no. 2.

**Figure 1:**
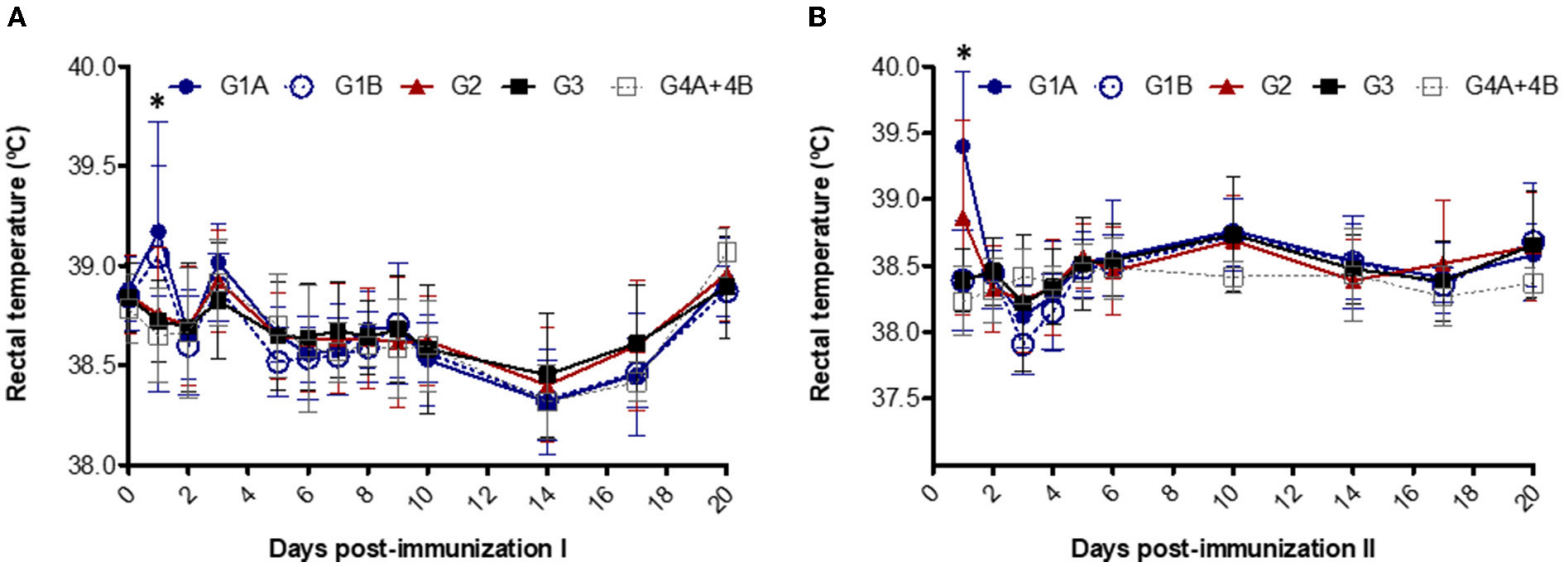
Rectal temperatures after the first **(A)** and second **(B)** immunizations. Immunized groups: Trichobovis administered by subcutaneous (SC; G1A) or intravulvar (IVU; G1B) routes and TrichGuard by SC route (G2). Non-immunized groups received PBS injections *via* SC (G3, G4A) or via IVU (G4B) routes. Each point represents the mean and standard deviation of the rectal temperature (°C; Y-axis) measured throughout the experimental time (X-axis; days post immunization). *Asterisk indicate significant differences between vaccinated and non-vaccinated groups.

Fever (> 39.5 °C) was detected in two animals from G1A (39.7 °C and 39.8 °C) and one animal from G1B (41 °C) on day 1 after the first immunization in study no. 1 (Figure 1A). After the booster, fever was observed in five animals from G1A (39.6 °C - 40.2 °C) and three animals from G2 (39.7 °C – 40.9 °C) on day 1 (Figure 1B). Significant differences were detected between non-vaccinated groups vs. G1A and G1B on day 1 after the first immunization and vs. G1A and G2 after booster (*P* < 0.05–0.0001; two-way ANOVA-Bonferroni post-test).

**Figure 2:**
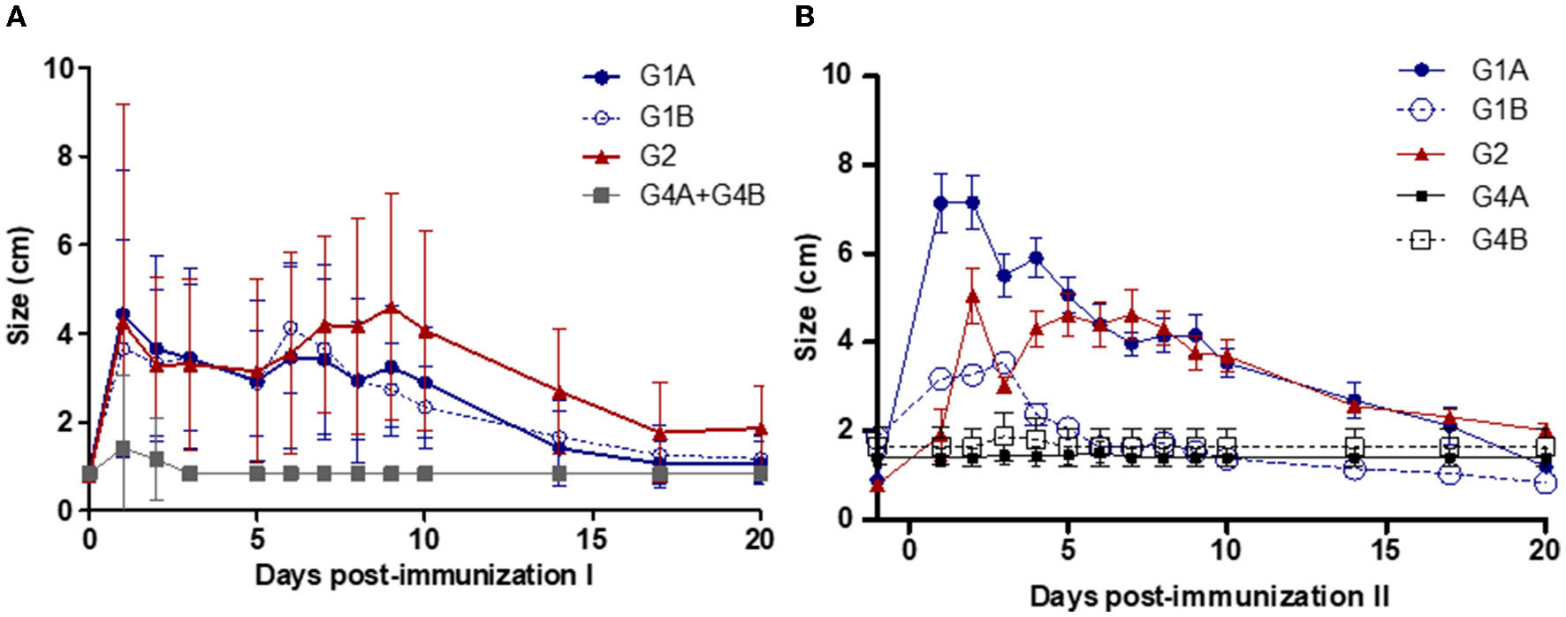
Local reaction sizes after the first **(A)** and second **(B)** immunizations. Immunized groups: Trichobovis administered by subcutaneous (SC; G1A) or intravulvar (IVU; G1B) routes and TrichGuard by SC route (G2). Non-immunized groups received PBS injections *via* SC (G4A) or *via* IVU (G4B) routes. Each point represents the mean and standard deviation of the local reaction sizes (cm; Y-axis) measured at different sampling times (X-axis; days post-immunization).

Local reactions were detected at the point of vaccination in more than 70% of the vaccinated animals after the first immunization and in all animals after the booster in study no. 1 (Table 1). None of the non-vaccinated animals developed local reactions. Local subcutaneous reactions consisted of mild cutaneous oedema in the vaccination site that evolved, turned hard, and almost disappeared on day 20 post-immunization (Figure 2). For local reactions after intravaginal booster (G1B), an inflammatory reaction was detected in the vulvar lip where the vaccine was inoculated, which resolved in 2 weeks. After the first immunization, significantly larger local reaction sizes were observed in the G1A (on days 1–3, 6–7 and 9), G1B (on days 1, 3, 6–7) and G2 (on days 1, 3–10) compared to the non-vaccinated groups (*P* < 0.05–0.0001; two-way ANOVA-Bonferroni post-test). After the booster, significant differences were observed in G1A (on days 1–10 after second immunization), G1B (on days 1–3) and G2 (on days 2–10) compared to the non-vaccinated groups (*P* < 0.05–0.0001; two-way ANOVA-Bonferroni post-test).

### *T. foetus* clearance

**Figure 3:**
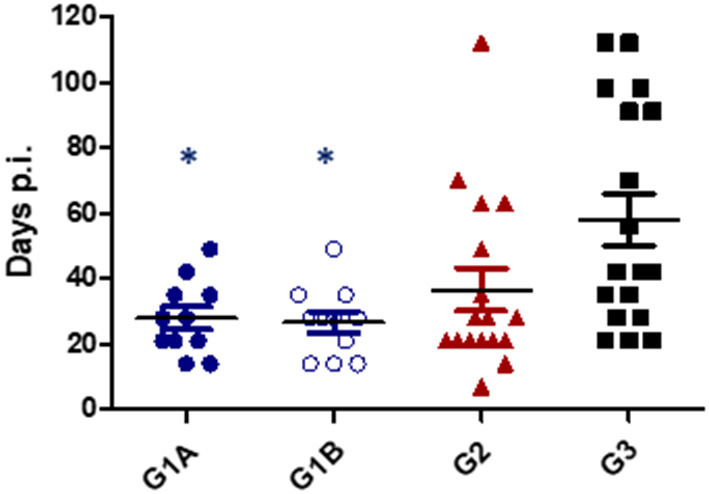
Length of *T. foetus* infection in the genital tract. Immunized/challenged groups: Trichobovis administered by subcutaneous (SC; G1A) or intravulvar (IVU; G1B) routes and TrichGuard by SC route (G2). Non-immunized/challenged group received PBS injections *via* SC (G3). All challenged cows were inoculated intravaginally with 2× 10^6^ *T. foetus* trophozoites. Data from each animal are presented as individual points. Horizontal lines represent median values for each group. Asterisks indicate significant differences in G1A and G1B vs. G3.

**Figure 4:**
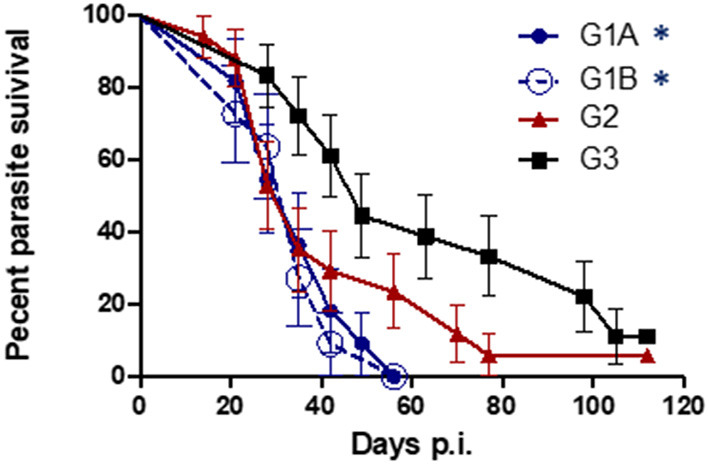
Kaplan-Meier curves. Survival proportions of the parasite in immunized/challenged groups: Trichobovis *via* subcutaneous (SC; G1A) or intravulvar (IVU; G1B) routes and TrichGuard vaccine by SC route (G2); and in non-immunized/challenged group (G3) throughout the experiment. All challenged cows were inoculated intravaginally with 2× 10^6^ *T. foetus* trophozoites. The vertical descent indicates the day p.i. on which an animal cleared the genital infection. Asterisks indicate significant differences in G1A and G1B vs. G3.

All challenged cows from study no. 1 were infected with *T. foetus* when CVM samples were cultured at days 1–7 post-challenge. The persistence of the infection was variable between groups, and 1/17 animals from G2 (TrichGuard) and 2/18 from G3 (positive group) remained infected at the end of the experiment (day 112 p.i.). In all animals immunized with Trichobovis vaccine (G1A and G1B groups), day 49 was the last post-challenge day of positive culture. Regularly negative cultures were observed in CVM samples taken from days 56 to 112 p.i. in the G1A and G1B groups. The average number of days of positive *T. foetus* isolation was shorter in the vaccinated groups G1A (28 ± 10.76 days), G1B (26.73 ± 10.26 days) and G2 (36.65 ± 25.92 days) compared to the non-vaccinated/challenged group (60.12 ± 32.57 days) (Figure 3). However, significant differences were observed only when the groups immunized with the new vaccine (G1A and G1B) were compared to the non-vaccinated/challenged group (G3) (*P* < 0.01, two-way ANOVA-Bonferroni post-test). No significant differences were found between the commercial vaccine (TrichGuard; G2) and non-vaccinated/challenged groups (G3) (*P* > 0.05, two-way ANOVA-Bonferroni post-test). Significantly lower parasite survival time in the genital tract was observed in Groups G1A and G1B vs. G3 (*P* < 0.01, log-rank test). No significant differences (*P* > 0.05) were observed in the survival curve between Group G2 (TrichGuard) and the non-vaccinated/challenged group (G3) (Figure 4).

### *T. foetus*-specific IgG and IgA responses

**Figure 5:**
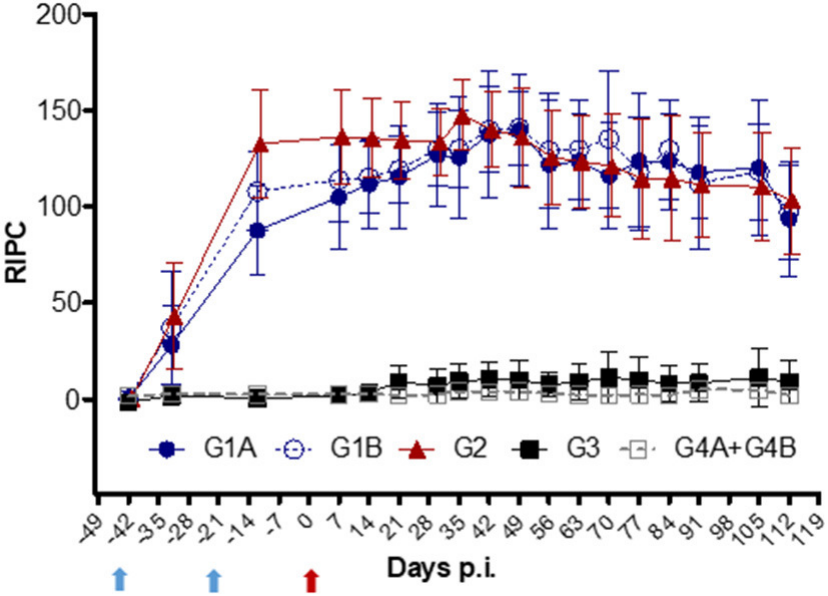
Kinetics of anti-*T. foetus* IgG levels measured by ELISA in sera. Immunized/challenged groups: Trichobovis *via* subcutaneous (SC; G1A) or intravulvar (IVU; G1B) routes, TrichGuard by SC route (G2). Non-immunized/challenged animals (G3) and non-immunized/non-challenged (G4A+G4B) groups. All challenged cows were inoculated intravaginally with 2× 10^6^ *T. foetus* trophozoites and non-challenged cows with PBS. The average IgG levels measured throughout the experimental time (X-axis; days p.i.) are represented in the graph using the relative index percent (RIPC) value (Y-axis). Error bars represent the standard deviation. Cows were vaccinated with two doses at 21-day intervals. Blue arrows highlight the day for immunization I (day−42) and the booster (day−21), and the red arrow highlights the day of the challenge (day 0).

IgG kinetics were similar for all vaccinated groups (G1A, G1B, and G2) (Figure 5). A significantly higher increase in IgG levels was observed in all vaccinated animals 10 days after the first immunization (day−32 p.i.) compared to the control groups (G3 and G4A+G4B; *P* < 0.0001, two-way ANOVA-Bonferroni post-test). The maximum levels were reached on day 35 p.i. and maintained at high levels until the end of the experiment (day 112 p.i.). The IgG values of non-vaccinated/challenged (G3) group remained close to uninfected levels throughout the experiment (*P* > 0.05, two-way ANOVA-Bonferroni post-test).

**Figure 6:**
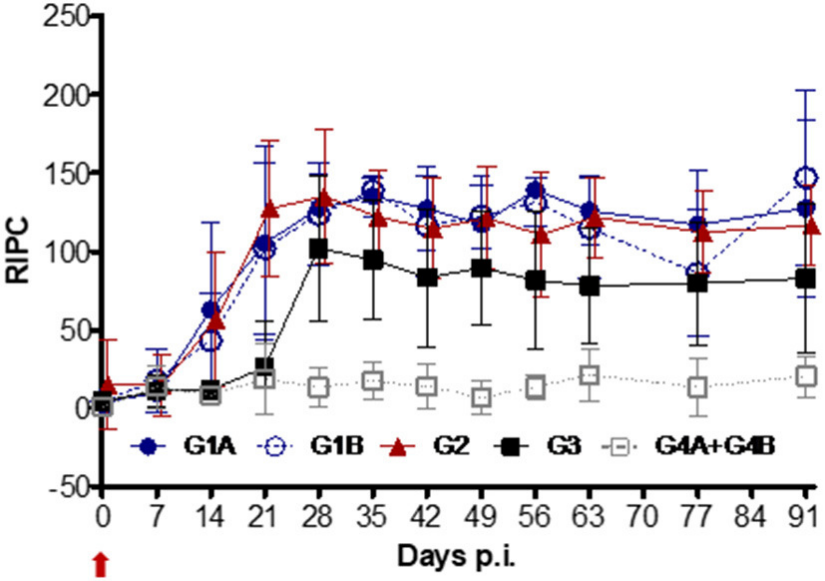
Kinetics of anti-*T. foetus* IgA levels measured by ELISA in CVM. Immunized/challenged groups: Trichobovis *via* subcutaneous (SC; G1A) or intravulvar (IVU; G1B) routes, TrichGuard vaccine by SC route (G2). Non-immunized/challenged (G3) and non-immunized/non-challenged (G4A+G4B) groups. All challenged cows were inoculated intravaginally with 2× 10^6^ *T. foetus* trophozoites and non-challenged cows with PBS. The average IgA levels measured throughout the experimental time (X-axis; days p.i.) are represented in the graph using the relative index percent (RIPC) value (Y-axis). Error bars represent the standard deviation. Day 0 is the day of the challenge (red arrow).

Before the challenge, no significant differences in IgA levels were observed in the vaccinated vs. non-vaccinated groups (data not shown). After challenge, a significant increase in IgA levels was found in all vaccinated groups (G1A and G2 from day 14 p.i. and G1B from day 21 p.i.) and the positive control (G3) (from day 28 p.i.) compared to the non-challenged group (G4A+G4B) (*P* < 0.001; ANOVA-Bonferroni post-test) (Figure 6). These levels remained significantly elevated until day 91 p.i. When IgA levels from the vaccinated groups were compared to the non-vaccinated/challenged group (G3), significantly higher IgA levels were observed in G1A (on days 14, 21, 35, 42, 56–91 p.i.), G1B (on days 21, 35, 63, 91 p.i.) and G2 (on days 14-28, 63-91 p.i.) (*P* < 0.05–0.0001; two-way ANOVA-Bonferroni post-test).

### Correlation between IgA and parasite clearance

Pearson correlation analysis showed significant values only for the groups immunized by SC route with Trichobovis (G1A) and TrichGuard (G2). Specifically, a significant negative correlation between IgA levels and the period of genital infection was observed in G1A on days 14 (*P* < 0.01) and 21 p.i. (*P* < 0.05) and G2 on days 28 (*P* < 0.01), 35 and 42 p.i. (*P* < 0.05). The strongest association was found in G1A on day 14 (*r* = −0.74) and in G2 on day 35 p.i. (*r* = −0.71).

### Reproductive effects of Trichobovis under field conditions

When the efficacy of Trichobovis was tested in a naturally infected herd (study no. 2), the calving intervals were significantly reduced by 45.2 days (*P* < 0.05) in the vaccinated group (group A) (Table 2). The percentage of calves born was nearly 8.7% higher (*P* = 0.1) but the differences were not significant. Calves were born 28 days earlier (*P* < 0.05) in group A compared to the placebo group (group B). Specifically, 73.3% (11/15) of the births occurred early in the calving season in group A, and 61.5% (8/13) of the calves from group B were born late (*P* > 0.05).

**Table 2:** Comparison of the reproductive data between animals vaccinated with Trichobovis group (A) and Placebo group (B).

**Parameter**	**Trichobovis group (A)**	**Placebo group (B)**	**Difference (A vs. B)**
Calving interval (mean ± SD)	408.06 ± 44.3 days	453.25 ± 60.6 days	−45.18 days*
Calving rate (born calves/mated cows)	15/23 (65.2%)	13/23 (56.5%)	+ 8.7% calves/year
Calving date (mean ± SD)	19 March ± 42.9 days	16 April ± 48.6 days	−28 days*
Early calf born rate^a^	11/15 (73.3%)	5/13 (38.5%)	+34.8% early calves/year

a Calf births occurred early in the calving season.

The asterisks indicate statistically significant differences between both groups.

## Discussion

Vaccination could be an essential component of any control program against bovine trichomonosis since there is no effective treatment and control measures are often expensive and difficult to implement. The main objective of vaccines against bovine trichomonosis is to eliminate *T. foetus* infection from the reproductive tract of the cow before foetal loss occurs. Whole-parasite-based vaccines (22–26, 30, 31, 35–39), vaccines formulated with membrane antigens of *T. foetus* or subunit vaccines (29, 35, 40–42) have been developed. Additional work has been also done to identify *T. foetus* surface antigens such as TF1.17 and TF190 (29, 42). In several of these studies, a shorter time of genital infection and a higher percentage of pregnant females have been reported. Although numerous studies have been carried out, there are only two inactivated vaccines against bovine trichomonosis available on the market: TrichGuard (Boehringer Ingelheim) and Tricovac (Laboratorio Biológico, Tandil) commercialized in America and Argentina, respectively. Several studies have showed that the commercial vaccine TrichGuard has been shown to be effective in reducing the duration of genital infections in vaccinated heifers, improving conception rate at first service, calving rates and reducing reproductive losses (22–26). In this work, a new vaccine formulation was developed capable to reduce the genital infection time with higher efficacy than the commercial vaccine (TrichGuard). The vaccine formulation was based on *T. foetu*s trophozoites inactivated by lyophilisation and Quil-A-adjuvanted. Whole parasite cells were used as vaccine antigen because they helped elicit a specific immune response that inhibited trichomonad adherence to bovine vaginal epithelial cells. Bovine serum antibodies to whole-cell *T. foetus* antigens immobilized and agglutinated trophozoites to enhance bovine complement and neutrophil-mediated killing of *T. foetus* (43). Most of the experimental and commercial vaccines were also based on whole killed cells (25, 31, 35), but chemical agents, such as merthiolate or formaldehyde (25, 30, 31, 35), were used as inactivation methods. Because of their chemical properties, the replacement of these products is advisable because they interact and modify immunogenic epitopes and may be toxic and carcinogenic (31, 44, 45). Lyophilisation was used as a non-hazardous and low-cost method to inactivate *T. foetus* trophozoites and preserve the parasite proteins because the temperature used was maintained below the denaturation of immunogenic epitopes, which provided a stable and sterile vaccine composition. Our vaccine formulation also included the Quil-A saponin as an adjuvant to increase the antigenic efficacy of the antigen. Quil-A saponin was selected because it may be administered *via* systemic and mucosal routes and its potential to enhance Th1 and Th2 responses (46). Quil-A saponins were previously used in vaccines against *T. foetus* (30, 41, 47) and other parasites (48), and promising results were obtained.

We used a well-established bovine model of genital *T. foetus* infection as proof-of-concept to evaluate the safety, efficacy and immunogenicity of the new vaccine formulation. Trichobovis may be considered safe in cattle because no adverse general reactions or clinical signs were observed, and only local reactions in the vaccination area were found. Efficacy was studied by determining the time of infection in the genital tract. Positive cultures from cattle infected with 10^6^-10^7^ *T. foetus* trophozoites are generally observed for longer than 70 days (26, 30, 31). The most remarkable result was that Trichobovis reduced the mean difference in the duration of infection by 32 days compared to the non-vaccinated/challenged group. The new vaccine was able to stimulate earlier parasite clearance than the commercial vaccine because no significant differences in the duration of the infection were observed between TrichGuard and the positive control group, which is consistent with a recent study using the same bovine model (26). Two doses of our formulation reduced the time of parasite clearance with higher or similar efficacy than other experimental vaccines using three doses of formalin *T. foetus* trophozoites and adjuvanted with Quil-A (duration of the genital infection = 41 days) or saponin plus aluminum hydroxide (= 27 days) (30). The vaccinated females remained infected for 3 to 4 weeks to a maximum of 7 weeks in other experiments with natural or experimental challenges (22, 24, 25, 29, 47, 49). The use of a different protocol for parasite inactivation (lyophilisation) in combination with Quil-A as an adjuvant may have contributed successfully to the results obtained in our study. This difference may be associated with the conservation of antigen epitopes during lyophilisation.

The importance of IgG and IgA in the elimination of the parasite from the female reproductive tract due to their opsonising properties was described previously (29, 41). The new vaccine stimulated high serum IgG and genital IgA levels and generated an IgG booster effect after the second vaccine dose in the present study, and these results were similar to the commercial vaccine. The elimination of the infection in vaccinated animals occurs from the moment that the IgA values are high (29, 47). The IgA levels in our study were associated with significantly earlier clearance of *T. foetus* from the genital tract in the vaccinated groups. Notably, a negative correlation was found on day 14 p.i. in the group immunized with Trichobovis subcutaneously, but this association was observed later (on day 35 p.i.) in the group vaccinated with the commercial vaccine. These results demonstrated that Trichobovis was capable of eliciting an effective mucosal response to eliminate the parasite from the genital tract earlier than the commercial vaccine. The long-term protection provided by our vaccine is unknown and further studies are necessary.

The route of administration of the vaccine may be a critical factor for improving *T. foetus* vaccine efficacy. In a previous study, an increase in serum antibody levels was observed when a formaldehyde-fixed whole cell-based vaccine with an oleaginous adjuvant was inoculated into the vaginal submucosa (36). A single instillation of formalin-fixed *T. foetus* cells significantly shortened *T. foetus* infection time vs. non-vaccinated animals (45 vs. 93.75 days) (31). In contrast, other studies using two doses of formalin-fixed parasites applied systemically and a third dose delivered intravaginally did not conclude that mucosal application improved vaccine efficacy (30, 35, 37, 38). When we compared SC or IVU immunization routes, the results were rather similar, and the genital route did not reinforce mucosal immunity. Both routes primed a mucosal IgA response, and no significant differences in the duration of genital parasite infection were found. The subcutaneous route may be more convenient for reasons of comfort, safety and welfare in the management of beef cattle.

Reproductive losses in bovine trichomonosis occur from approximately 70 and 90 days after infection (10, 50). A previous study demonstrated that early clearance of *T. foetus* improved pregnancy rates (26), but there is limited information of the impact of the vaccines against bovine trichomonosis on the reproductive performance under natural challenge conditions (24, 25). All animals immunized with the new vaccine in the first experiment cleared the infection before day 56 p.i. In light of these encouraging results, a second study was performed to evaluate the protection of Trichobovis against the reproductive effects of bovine trichomonosis in cows from an infected herd according to the epidemiological aspect of the target disease. Our findings suggest that the new vaccine protected against early pregnancy losses because the calving interval was significantly shortened and calves were born earlier. These results might have a high impact on the efficiency of a suckler herd because calves born early in the calving season will have heavier weaning weights and the feeding costs will be lower. Unfortunately, weaning percentages and weaning weights of the calves were not available in our study. Another remarkable result was that the reduction of the calving intervals will decrease the losses from the cost of maintaining non-productive cows (i.e., cows not pregnant at the end of the breeding season or that fail to deliver a live calf before the next breeding season). Further studies are required to evaluate the financial benefits of Trichobovis.

The vaccinated cows from our study had a higher calving rate but the differences were not significant compared with the placebo group, which may be due to the low number of animals involved in this study. Moreover, during the long mating period (more than 120 days) used in the management of the “Asturiana de la Montaña” breed, natural immunity would have mounted after clinical infection, and non-vaccinated cows would have recovered from infection, become pregnant and calved later in the calving season. In fact, non-vaccinated cows showed a higher percentage of late-born calves than the vaccinated group. Therefore, further studies using a short calving season (60–90 days) must be performed in a large cattle population to provide additional data on the effectiveness of the new vaccine under field conditions.

We highlight that vaccination effects highly depend on the epidemiological and management situation of the herd. A vaccine against bovine trichomonosis may be recommended in the vaccination plan (two parenteral vaccine doses before mating) of herds at greater risk for introduction of *T. foetus* based on relatively high local prevalence of the infection or the use of management practices that contribute to the introduction of the agent into the herd and cattle-to-cattle transmission, particularly factors related to animal movement, pasture management, or biosecurity measures.

## Conclusions

The findings of this study indicate that Trichobovis vaccine was well tolerated by cattle and stimulated humoral and mucosal immunity against *T. foetus* to reduce the length of the parasite genital infection in cows under experimental challenge. Under natural infection situations, this effect translated into shortened calving intervals and earlier calving dates, which improved the reproductive performance. These results obtained should be considered a significant indicator of the benefit of vaccination in the cattle population and conditions in which the vaccine will ultimately be used.

## Data availability statement

The raw data supporting the conclusions of this article will be made available by the authors, without undue reservation.

## Ethics statement

The animal study was reviewed and approved by the Animal Research Ethics Committee (Reference Number PROAE 13/2014) of the Community of Principado de Asturias, Spain. The field study was performed according to the protocol with reference TRIVAC-FR-RD-1 authorized by the Spanish Agency for Medicines and Health Products (AEMPS) with authorization number 391/ECV and PIV reference 181/PIV. Written informed consent was obtained from the owner for the participation of their animals in this study.

## Author contributions

Conceptualization: LO-M, EC-F, DM-G, EP-C, AP-R, and KO. Methodology: RS-S, SR-M, and AR-T. Data analysis: EC-F and AP-R. Writing and original draft preparation: EC-F. Writing—review and editing: RS-S, JR-C, LO-M, and EC-F. Visualization supervision: RS-S and EC-F. All authors have read and agreed to the published version of the manuscript.

## Funding

This study was supported by the PLATESA2 project (S2018/BAA-4370) of the Community of Madrid and the collaborative agreement between CZ VETERINARIA and SALUVET-Innova to develop of an inactivated vaccine against *Tritrichomonas foetus* in cattle.

## Conflict of interest

Authors DM-G, EP-C, and AP-R were employed by CZ Vaccines S.A.U., ZENDAL Group. The remaining authors declare that the research was conducted in the absence of any commercial or financial relationships that could be construed as a potential conflict of interest.

## Publisher's note

All claims expressed in this article are solely those of the authors and do not necessarily represent those of their affiliated organizations, or those of the publisher, the editors and the reviewers. Any product that may be evaluated in this article, or claim that may be made by its manufacturer, is not guaranteed or endorsed by the publisher.
